# Supplementary material for: Occurrence and Determination of Carotenoids and Polyphenols in Different Paprika Powders from Organic and Conventional Production
Source: Molecules. 2021 May 17;26(10):2980. doi: 10.3390/molecules26102980 (PMC8156602; doi:10.3390/molecules26102980)
Supplement: Supplementary file 1 [file molecules-26-02980-s001.zip › molecules-1204369-supplementary.pdf]

Table S1. Chemical structures of the major compounds identified

Compound	Structure
***beta*-carotene**	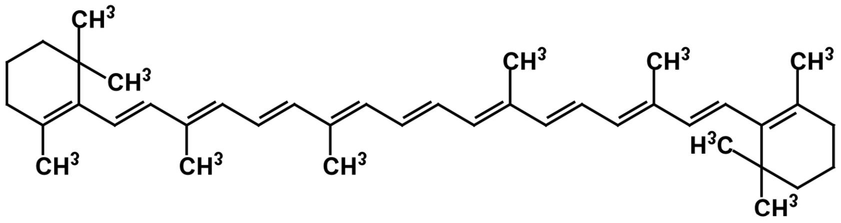
***alpha*-carotene**	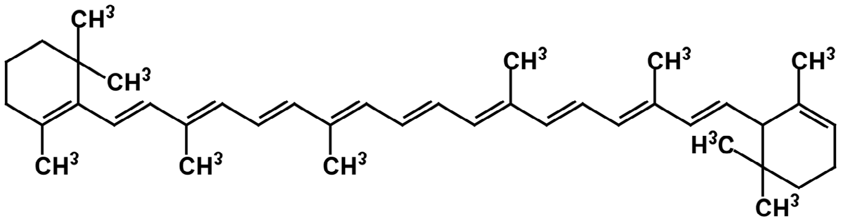
**capsorubin**	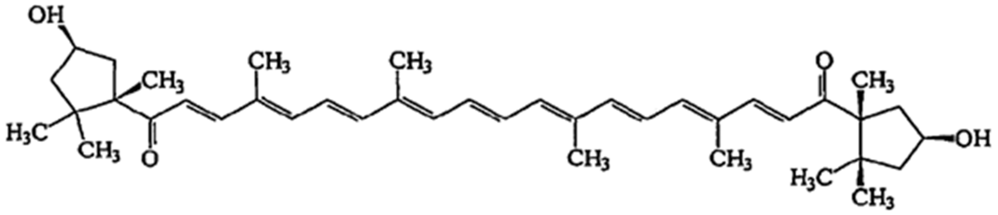
**cryptixanthin**	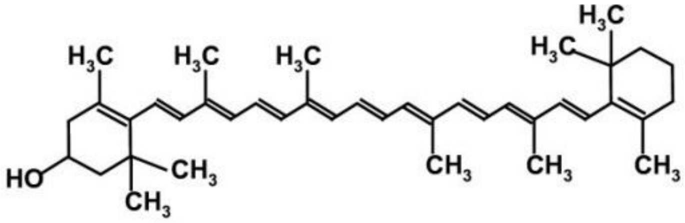
**lutein**	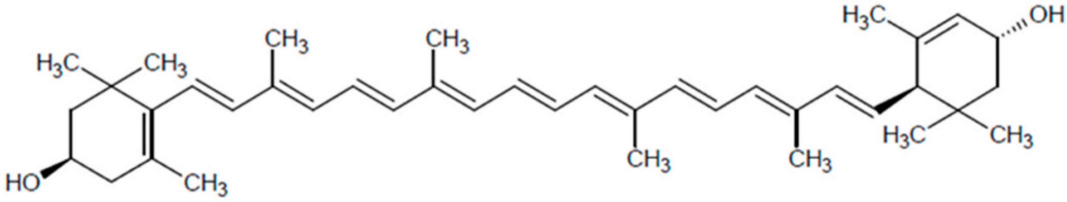
**zeaxanthin**	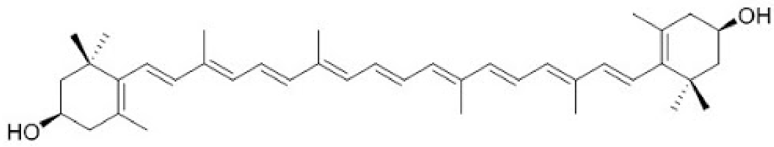
**capsaicin**	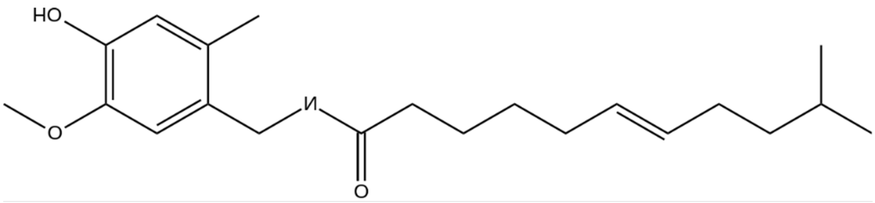
**gallic acid**	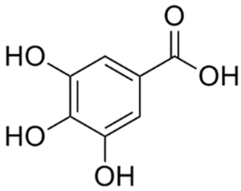

**chlorogenic acid**	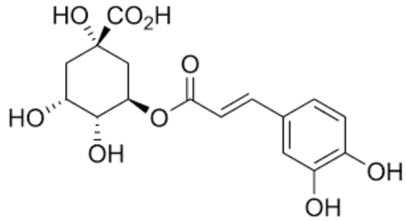
**caffeic acid**	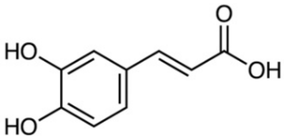
***p*-coumaric acid**	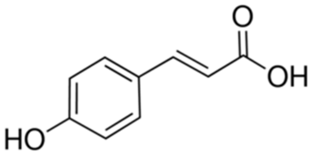
**ferulic acid**	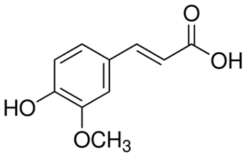
**quercetin-3-*O*-rutinoside**	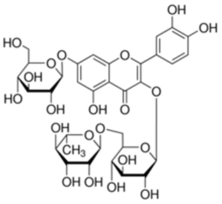
**myricetin**	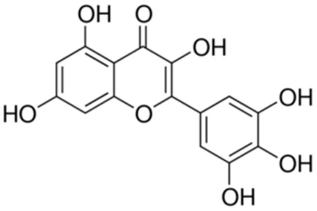
**quercetin**	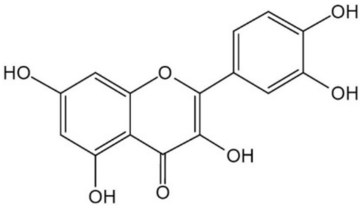
**quercetin-3-*O*-glucoside**	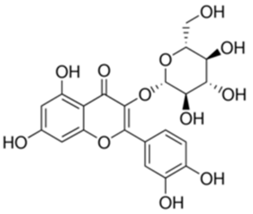
**kaempferol**	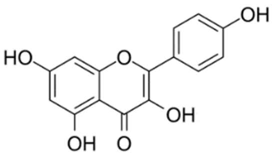

Table S2. The content of total and major carotenoids in different good food sources

Product	**total carotenoids**	***beta*-carotene**	***alpha*-carotene**	**lutein**	**zeaxanthin**
	**mg/100g FW**				
Carrot	5.81 - 10.20[3]	4.16 -8.84[3,8]	1.34 - 4.96[3,8]	0.17-0.51[2,3,4]	in[4]
Pumpkin	36 [1]	0.2-17.2 [1,8]	0.4-1.5 [1]	0.6 3 [1,8]	-[8]
Spinach	17.66-22.63[10]	3.10-4.81[8]	-[8]	5.93 - 8.26[2,4,8]	in[4,8]
Broccoli	20.77[15]	0.29-1.75[8]	-[8]	0.71-3.30[2,4,8]	-[8]
Apricot	1.76 - 22.2[12]	0.59-3.8[8]	0-0.04[8]	0.12-0.19[8]	0-0.04[8]
Red Tomato	4.41-7.85[6]	0.1–1.5[5,8]	0–0.002[5]	0.09-0.21[5,8]	in[8]
Cherry tomato	6.33-11.2[7]	0.24-0.37[7]	-	-	0.02-0.06[7]
Red pepper	3.33[14]	1.4-2.39[8]	0-0.29[8]	0.25-8.51[2.4,8]	0.59 - 1.35[4,8]
Red Jalapeno pepper	23.4 - 43.8[11]	0.38-8.58[8]	0.01-0.17[8]	0.84[8]	-[8]
	**mg/100g DW**				
Yellow Paprika	20.72[13]	*3.19[13]	0.57[13]	14.37[13]	0.264[13]
Orange Paprika	97.53[13]	*9.74[13]	2.14[13]	31.9[13]	44.9[13]
Red Paprika	45.77[13]	*19.34[13]	0.24[13]	2.14[13]	6.93[13]
Jalapeno pepper	106.5[9]	7.7[9]	-[9]	-[9]	10.4[9]

FW- fresh weight, DW - dry mass; in = included with lutein; -: not included in the references;
*13Z-β-carotene, E-β-carotene and 9Z-β-carotene sum

Table S3. Total polyphenol content in different vegetables

Product	Total polyphenol mg/100g	
	Fresh weight	Dry mass
Tomato	62	1422-1564
Carrot	156	1400-1582
Italian Cabbage	108	1573-1670
Onion	150	1221-1483
Broccoli	290	1621-2363

Source: [16]

Table S4. Amounts of major flavonols of selected vegetables

Product	Flavonols mg/100g FW		
	Quercetin	Myricetin	Kaempferol
Sweet potato leaves (green)	14.38	3.88	-
Sweet potato leaves (purple)	26.68	15.58	-
Spinach	0.042	1.96	0.056
Onion	2.6	0.021	0.056
Onion - leaves	2.58	-	0.52

. -: not included in the references Source: [17]

1. Niewczas J., Szweda D., Mitek M. The content of selected pro-healthful components in winter squash (*Cucurbita maxima*) fruits. *Żywność, Nauka. Technologia. Jakość,* **2005**, 2 (43) Supl., 147 – 155

2. Yoon G.-A., Yeum K.-J., Cho Y.-S., Chen O., Tang G., Blumberg J.B., Russell R.M., Yoon S., Lee-Kim Y. Carotenoids and total phenolic contents in plant foods commonly consumed in Korea. *Nutr Res Pract.*, **2012**, 6(6): 481–490. doi: 10.4162/nrp.2012.6.6.481

3. Bozalan N.K., Karadeniz F. Carotenoid Profile, TotalPhenolic Content, and Antioxidant Activity of Carrots, *International Journal of Food Properties*, **2011**, 14:5,1060-1068, DOI: 10.1080/10942910903580918 https://www.tandfonline.com/doi/pdf/10.1080/10942910903580918

4. El-Sayed M. A.-A., Humayoun A., Khalid Z., Rashida A Dietary Sources of Lutein and Zeaxanthin Carotenoids and Their Role in Eye Health. *Nutrients*, **2013,** 5(4): 1169–1185. doi: 10.3390/nu5041169

5. Martí R., Roselló S., Cebolla-Cornejo J. Tomato as a Source of Carotenoids and Polyphenols Targeted to Cancer Prevention. *Cancers (Basel,* **2016,** 8(6): 58. doi: 10.3390/cancers8060058

6 Mendelova A., Fikselova A., Mendel L. Carotenoids and lycopene content in fresh and dried tomato fruits and tomato juice. *Acta Univ. Agric. Silvic. Mendelianae Brun.* **2013**, 61, 1329-1337 DOI: 10.11118/actaun201361051329

7. Pék Z., Szuvandzsiev P., Daood H., Nemenyi A., Helyes L. Effect of irrigation on yield parameters and antioxidant profiles of processing cherry tomato. *Central European Journal of Biology*, **2014,** 9. 383. doi: 10.2478/s11535-013-0279-5.

8. Elvira-Torales L.I., García-Alonso J., Periago-Castón M.J. Nutritional Importance of Carotenoids and Their Effect on Liver Health: A Review *Antioxidants (Basel)* **2019** Jul; 8(7): 229 doi: 10.3390/antiox8070229

9 Arimboor R., Natarajan R.B., Menon K.R., Chandrasekhar L.P., Moorkoth V. Red pepper (*Capsicum annuum*) carotenoids as a source of natural food colors: analysis and stability—a review *J Food Sci Technol.* **2015** 52(3): 1258–1271. doi: 10.1007/s13197-014-1260-7

10. Kidmose U., Knuthsen P., Edelenbos M., Justesen U., Hegelund E. Carotenoids and flavonoids in organically grown spinach (*Spinacia oleracea* L) genotypes after deep frozen storage. *Journal Of The Science Of Food And Agriculture* **2001,** 81 (9), 918-923 https://doi.org/10.1002/jsfa.902

11. Wall M.M, Waddell C.A., Bosland P.W. Variation in β-Carotene and Total Carotenoid Content in Fruits of *Capsicum HortScience* **2001,** 36 (4):746–749

12. Zhou W., Niu Y., Ding X., Zhao S., Li Y., Fan G., Zhang S., Liao K. Analysis of carotenoid content and diversity in apricots (Prunus armeniaca L.) grown in China *Food Chem* **2020,** 330, 1-12, https://doi.org/10.1016/j.foodchem.2020.127223

13. Park SY., Choi S.R., Lim S.H., Yeo Y., Kweon S.J Bae Y.S., · Kim K.W., Im K.H., Ahn S.K, Ha S.H, Park S.U., Kim J.K. Identification and Quantification of Carotenoids in Paprika Fruits andCabbage, Kale, and Lettuce Leaves *J Korean Soc Appl Biol Chem* **2014,** 57(3), 355–358

14. Sahabi M., Shehu R.A, Saidu Y., Abdullahi A.S Screening for Total Carotenoids and β-Carotene in Some Widely Consumed Vegetables in Nigeria *Nigerian Journal of Basic and Applied Science* **2012**, 20(3): 225-227

15. Reis L.C.R., Oliveira V.R., Hagen M.E.K., Jablonski A., Flôres S.H., Rios A.O. Carotenoids, flavonoids, chlorophylls, phenolic compounds and antioxidant activity in fresh and cooked broccoli (Brassica oleracea var. Avenger) and cauliflower (Brassica oleracea var. Alphina F1) *Food Science and Technology* **2015,** 63 (1), 177-183

16. Cieślik E., Gręda A., Adamus W. Contents of polyphenols in fruit and vegetables *Food Chemistry* **2006**, 94, 135–142

17. Chu YH., Chang CL., Hsu HF. Flavonoid content of several vegetables and their antioxidant activity *Journal of the Science of Food and Agriculture* **2000,** 80:561-566
